# Phenolic compounds from Rosemary (*Rosmarinus officinalis* L.) attenuate oxidative stress and reduce blood cholesterol concentrations in diet-induced hypercholesterolemic rats

**DOI:** 10.1186/1743-7075-10-19

**Published:** 2013-02-02

**Authors:** Milessa S Afonso, Ana Mara de O Silva, Eliane BT Carvalho, Diogo P Rivelli, Sílvia BM Barros, Marcelo M Rogero, Ana Maria Lottenberg, Rosângela P Torres, Jorge Mancini-Filho

**Affiliations:** 1 Department of Food and Experimental Nutrition, Faculty of Pharmaceutical Sciences, University of São Paulo, 05508-900, São Paulo, Brazil; 2 Department of Clinical and Toxicological Analyses, Faculty of Pharmaceutical Sciences, University of São Paulo, 05508-900, São Paulo, Brazil; 3 Department of Nutrition, School of Public Health, University of São Paulo, 01246-904, São Paulo, Brazil; 4 Lipids Laboratory (LIM-10), Faculty of Medical Sciences, University of São Paulo, 01246-000, São Paulo, Brazil

**Keywords:** Hypercholesterolemia, Oxidative stress, Polyphenols, *Rosmarinus officinalis*

## Abstract

**Background:**

Phenolic compounds combine antioxidant and hypocholesterolemic activities and, consequently, are expected to prevent or minimize cardiometabolic risk.

**Methods:**

To evaluate the effect of an aqueous extract (AQ) and non-esterified phenolic fraction (NEPF) from rosemary on oxidative stress in diet-induced hypercholesterolemia, 48 male 4-week old Wistar rats were divided into 6 groups: 1 chow diet group (C) and 5 hypercholesterolemic diet groups, with 1 receiving water (HC), 2 receiving AQ at concentrations of 7 and 140 mg/kg body weight (AQ70 and AQ140, respectively), and 2 receiving NEPF at concentrations of 7 and 14 mg/kg body weight (NEPF7 and NEPF14, respectively) by gavage for 4 weeks.

**Results:**

*In vitro*, both AQ and NEPF had remarkable antioxidant activity in the 2,2-diphenyl-1-picrylhydrazyl (DPPH^●^) assay, which was similar to BHT. *In vivo*, the group that received AQ at 70 mg/kg body weight had lower serum total cholesterol (−39.8%), non-HDL-c (−44.4%) and thiobarbituric acid reactive substance (TBARS) levels (−37.7%) compared with the HC group. NEPF (7 and 14 mg/kg) reduced the tissue TBARS levels and increased the activity of tissular antioxidant enzymes (superoxide dismutase, catalase and glutathione peroxidase). Neither AQ nor NEPF was able to ameliorate the alterations in the hypercholesterolemic diet-induced fatty acid composition in the liver.

**Conclusions:**

These data suggest that phenolic compounds from rosemary ameliorate the antioxidant defense in different tissues and attenuate oxidative stress in diet-induced hypercholesterolemic rats, whereas the serum lipid profile was improved only in rats that received the aqueous extract.

## Background

Atherosclerosis is a chronic inflammatory disease initiated by the subendothelial retention of low density lipoprotein (LDL) particles followed by their subsequent oxidation. The chemical modification of LDL particles induces the expression of adhesion molecules, such as vascular cell adhesion molecule-1 (VCAM-1) and intercellular adhesion molecule-1 (ICAM-1), on endothelial cells and smooth muscle cells, which, once activated, lead to cytokine secretion and contribute to the recruitment of monocytes and T cells to the arterial intima [1,2]. Macrophage colony-stimulating factor (MCSF) induces monocytes entering the plaque to differentiate into macrophages that display scavenger receptors on their surface. These receptors promote modified LDL uptake in a process that is not regulated by the intracellular lipid content, which ultimately leads to foam cell formation. Therefore, hemodynamic stress and the accumulation of lipids initiate an inflammatory process in the artery wall [3,4].

Moreover, the accumulation of cholesterol in erythrocytes, leukocytes, platelets and endothelial cells can lead to an increase in the concentration of reactive species [5,6] and a reduction in the antioxidant defense systems, such as catalase (CAT) glutathione peroxidase (GPx) and superoxide dismutase (SOD) enzyme activities [7]. This condition favors a disruption of the redox balance, contributing to the establishment of oxidative stress, which is involved in several metabolic disorders [8,9]. Thus, substances that combine antioxidant and hypocholesterolemic activities are expected to prevent or attenuate the cardiometabolic risk because the oxidative modification of LDL and its uptake by macrophages present in the arterial wall contribute to cardiovascular disease [10].

Phenolic compounds have been proven to be successful in attenuating hypercholesterolemia [11-13]. Moreover, these substances are known by their capacity to increase antioxidant enzyme activity and reduce free radical formation, effects that have been of interest to researchers as possible protective agents in diseases involving oxidative stress [7,10,14]. Among the herbal extracts reported to have antioxidant activity, rosemary (*Rosmarinus officinalis* L.) is one of the most widely commercialized plant extracts; it is used as a culinary herb for flavoring and as an antioxidant in processed foods and cosmetics [15]. Regarding its antioxidant and anti-inflammatory activities, studies have identified phenolic compounds obtained through several extraction methods, mainly carnosic (phenolic diterpene) and rosmarinic acids (caffeic acid and 3,4-dihidroxifenilactate ester) [16-18]. Although antioxidant compounds have been demonstrated to have beneficial effects on some parameters related to the cardiovascular risk, the role of rosemary phenolic compounds on hypercholesterolemia-induced oxidative stress has not been elucidated in the literature. Therefore, the aim of this study was to evaluate the effect of two different extracts, an aqueous extract and a non-esterified phenolic fraction, from rosemary (*Rosmarinus officinalis* L.) on the antioxidant status of the serum and tissues from diet-induced hypercholesterolemic rats.

## Methods

### Preparation of the aqueous extract and non-esterified phenolic fraction

Dried and powdered rosemary leaves (20 g) were extracted with distilled water (100 mL) at room temperature (23°C) for 1 h. The samples were centrifuged at 15,000 rpm for 10 min. The supernatant was filtered through qualitative filter paper, and distilled water was added to a total volume of 100 mL. The non-esterified phenolic fraction was obtained by homogenizing powdered, defatted rosemary leaves (1 g) with tetrahydrofuran (6 successive one-minute periods with 20 mL) at room temperature [19]. The supernatant was filtered with sodium sulfate anhydrous and vacuum-evaporated to dryness at 30°C. The dried mass was then resuspended in 5 mL of methanol and filtered through qualitative filter paper into a 5 mL volumetric flask, and methanol was added to achieve the final volume of 20 mL. The solutions were kept at −20°C until further analysis.

### Determination of total phenolic content

The total phenolics were determined by the Folin-Ciocalteau method [20]. The total phenolic content was expressed as milligrams of gallic acid equivalent (GAE) per gram of leaf.

### High performance liquid chromatography (HPLC) analysis

Rosmarinic and carnosic acids were identified and quantified by a modified version of the method described by Almela et al. [21] using HPLC with UV/Vis detection and a reversed-phase Luna C18 column (250 x 4.3 x 10 μm, Phenomenex, USA). The mobile phase consisted of 0.2% metaphosphoric acid (A) and acetonitrile (B) using a gradient program of 0–20 min 80% A, 20–30 min 60% A, and 30–40 min 0% A. The flow rate was 1 mL/min. The peaks for rosmarinic and carnosic acids, which have detection limits of 1.58 μg/mL and 1.46 μg/mL, respectively, were detected at 330 and 230 nm, respectively. The peaks were identified by comparing their retention times with those of standards (Sigma Aldrich, St Louis, MO, USA), which were run individually. Carnosic and rosmarinic acid calibration curves (R^2^ = 0.999 for both) were used for quantification.

### Determination of in vitro antioxidant activity using the β-carotene/linoleic acid system and DPPH^●^ assay

The β-carotene/linoleic acid co-oxidation system was conducted as previously described [22]. The antioxidant activity of the samples was also assessed by the 2,2-diphenyl-1-picrylhydrazyl (DPPH^●^) radical method [23]. The results were expressed as μmol equivalents BHT/g of sample.

### Animals and diet

Male Wistar rats (*Rattus norvegicus*, var. albinus) (101.3 ± 0.4 g) were obtained from the Animal Laboratory of the Faculty of Pharmaceutical Sciences at the University of São Paulo. A total of forty-eight rats (n = 8 per group, 4 weeks old) were kept in a room at an ambient temperature of 22 ± 2°C and a relative humidity of 55 ± 10% under a 12-h light/12-h dark cycle (lights on at 0700 h). All procedures performed on animals were approved by the Ethics Committee on Animal Experimentation of the Faculty of Pharmaceutical Sciences, University of São Paulo (protocol number 174/2008), according to the guidelines of the Brazilian College on Animal Experimentation. The animals were randomly distributed into six groups: C (chow diet, receiving distilled water), HC (hypercholesterolemic diet, receiving distilled water), AQ 70 and 140 (hypercholesterolemic diet, treated with 70 and 140 mg aqueous extract/kg body weight/d, respectively), and NEPF 7 and 14 (hypercholesterolemic diet, treated with 7 and 14 mg of non-esterified phenolic fraction/kg body weight/d, respectively).

To induce hypercholesterolemia, the chow diet (AIN-93M Nuvilab® CR-1, Nuvital, BRA) was enriched with 0.5% cholesterol and 0.25% cholic acid (Sigma Aldrich) (Table 1). Food and water were provided *ad libitum* throughout the experiment. The aqueous extract, non-esterified phenolic fraction and distilled water were administered daily by gavage (0.5 mL/100 g body weight) during the light cycle (at 1300 h) for 4 weeks. Food consumption and weight were monitored daily throughout the experiment. After 4 weeks, the rats were deprived of food for 12 hours and anesthetized with ketamine (90 mg/kg, Vetbrands, BRA) and xylazine (10 mg/kg, Vetbrands, BRA). All of the animals were killed in the morning between 08:00 a.m. and 12:00 p.m. The tissues were perfused with saline (0.9% w/v), collected and immediately frozen. The blood was collected from the abdominal aorta, and serum was obtained by centrifugation at 3,500 rpm at 4°C for 5 minutes. The serum and tissues were stored at −80°C until further analysis.

**Table 1:** **Composition of the experimental diets (g/kg diet)**^**a**^

**Parameters (g/kg diet)**	**Chow diet**	**Hypercholesterolemic diet**
Protein	220	220
Lipids	40	40
Fiber	80	80
Mineral mix^*^	100	100
Carbohydrates	560	560
Cholesterol	0	5
Cholic acid	0	2.5

^**a**^ Nuvilab® CR-1, Nuvital, BRA.

^*^Mineral mix: Iron 50 mg; zinc 60 mg; copper 10 mg; iodine 2 mg; manganese 60 mg; selenium 0.05 mg; cobalt 1.50 mg.

**Vitamin Mixture provided the following amounts (per kg of diet):** Vitamin A 12,000 UI; vitamin E 30 mg; vitamin K 3 mg; B vitamins 18 mg, niacin 60 mg; pantothenic acid 20 mg; folic acid 1 mg; biotin 0.05 mg, choline 600 mg.

Diets were isoenergetic (15 MJ/kg of diet) and given in pellet form.

### Extraction and esterification of hepatic lipids

Lipids from the hepatic tissue were obtained by the Folch method [24], and the fatty acid methyl ester content was determined according to the *American Oil Chemists Society* method Ce 2–66 [25].

### Fatty acid profile of hepatic tissue

The samples were analyzed by gas chromatography (Shimadzu, GC 17A, Kyoto, JAP) using a fused silica capillary column (100 m × 0.25 mm, SP-2560). After a 5 minute isothermal period, the temperature of the column was increased from 140°C to 240°C at a rate of 4°C/min and then kept constant for 20 minutes. The injector and detector temperatures were 250°C and 260°C, respectively. Helium was employed as the carrier gas at a flow rate of 1 mL/min, and a sample volume of 1 μL was injected at a split ratio of 1:200. The peaks were identified by comparing their retention times with those of the standards (Supelco 18919, USA), which were run at a split ratio of 1:50.

### Activity of tissue antioxidant enzymes

The tissular catalase (CAT), superoxide dismutase (SOD) and glutathione peroxidase (GPx) activities were determined as described by Beutler [26], McCord and Fridovich [27] and Sies et al. [28], respectively. All analyses were conducted using a spectrophotometer, and absorbances were monitored every 60 seconds for 6 minutes.

### Protein quantification

A spectrophotometric method [29] was used to determine the protein content in the serum and tissues analyzed.

### Determination of thiobarbituric acid-reactive substances (TBARS)

The method described by Ohkawa et al. [30] was employed. The absorbance was measured at 532 nm on a spectrophotometer. The TBARS concentrations were calculated using a standard curve for 1,1^′^,3,3^′^-(TEP) tetraethoxypropane (10^-4^ mol/L) and were expressed as μmol of malondialdehyde (MDA) per milligram of protein.

### Serum lipids

The serum total cholesterol (TC), triacylglycerol (TG), and high density lipoprotein cholesterol (HDL-c) levels were assessed using commercial enzymatic colorimetric *kits* (Labtest®, BRA). The non-high-density lipoprotein cholesterol (non-HDL-c) levels were calculated by subtracting the HDL-c levels from the total cholesterol levels [31].

### Statistical analysis

Statistical analyses were conducted using GraphPad Prism 4.0 for Windows (San Diego, CA, USA). The collected data were subjected to the Kolmogorov-Smirnov test to check for symmetry. The data were expressed as the mean and standard error, and the significance level was set at 0.05. Student’s t test was used to compare two independent samples (the C and HC groups). Three or more independent samples (treated or non-treated HC groups) were compared using a one-way analysis of variance (ANOVA) and Tukey’s test (p < 0.05).

## Results

### Characterization of the aqueous extract and non-esterified phenolic fraction from Rosmarinus officinalis

The total phenolic content in the extracts was 16.67 ± 0.40 mg GAE/g leaf in the AQ and 8.59 ± 0.31 mg GAE/g leaf in the NEPF. Regarding the major compounds, 1.87 ± 0.02 g of rosmarinic acid/100 g dry weight was detected in the AQ, and 5.71 ± 0.05 g of carnosic acid/100 g dry weight was detected in the NEPF; these levels are consistent with the results from other studies [18]. The antioxidant potential of the rosemary extracts were evaluated using the DPPH^●^ method, yielding 4601.29 ± 60.54 and 4752.80 ± 190.09 μmol BHT equivalents/g for the AQ and NEPF, respectively. All values were comparable with the BHT activity (4538.00 μmol/g), confirming that both extracts possess the hydrogen-donating capability to act as an antioxidant (Table 2). In the β-carotene-linoleate model system, AQ at 500 and 1000 μg showed an antioxidant activity of 113.25 ± 4.55 and 118.03 ± 3.88 μmol BHT equivalents/g, respectively. However, the antioxidant activity obtained with NEPF at 50 and 100 μg was approximately 10 times higher than the aqueous extract effect (929.35 ± 49.88 and 1035 ± 21.21 μmol BHT equivalents/g, respectively).

**Table 2:** ***In vitro *antioxidant activity of the aqueous extract and non-esterified phenolic fraction of rosemary**

**Samples**	**β-carotene/linoleic acid system**		**DPPH**^**●**^
	**antioxidant activity μmol BHT/g sample**		
**Aqueous Extract**	**500 μg**	**1000 μg**	
	113.25 ± 4.55^A^	118.03 ± 3.88^A^	4601.29 ± 60.54^A^
**Non-Esterified Phenolic Fraction**	**50 μg**	**100 μg**	
	929.35 ± 49.88^B^	1035.00 ± 21.21^B^	4752.80 ± 190.09^A^

Butylated hydroxytoluene (BHT) antioxidant activity = 4538.00 μmol/g.

The results are expressed as the means ± S.E.M. (n = 3).

### Body weight, food intake and weight gain

The *in vivo* experiments showed that the mean values of weight gain and food intake were not significantly different among the groups and therefore the food efficiency ratio (FER) was similar (Table 3), suggesting that the phenolic compounds present in rosemary did not impair the nutrient intake (Table 3).

**Table 3:** Weight gain, food intake, food efficiency ratio (FER) and lipid profile of the experimental groups

	**C**	**HC**	**AQ 70**	**AQ 140**	**NEPF 7**	**NEPF 14**
**Weight Gain (g)**	167.64 ± 13.41	179.21 ± 24.13ª	175.95 ± 16.22ª	174.06 ± 25.68ª	175.53 ± 17.75ª	179.67 ± 24.71ª
**Food intake (g/day)**	20.24 ± 2.82	21.85 ± 3.77ª	21.52 ± 3.38ª	21.32 ± 3.20ª	21.79 ± 3.53ª	22.05 ± 4.63ª
**FER**	0.38	0.37	0.37	0.37	0.37	0.39
**TC (mg/dL)**	61.30 ± 6.46	178.54 ± 33.31ª^*^	107.47 ± 32.42^b^	128.85 ± 23.71^ab^	139.01 ± 38.25^a^	143.01 ± 37.92^a^
**Non-HDL-c (mg/dL)**	26.46 ± 1.76	152.00 ± 14.67ª^*^	84.47 ± 12.78^b^	103.70 ± 10.03^ab^	120.60 ± 17.46^a^	127.20 ± 18.59^a^
**HDL-c (mg/dL)**	38.15 ± 6.49	21.36 ± 3.47ª^*^	25.04 ± 2.57ª	25.28 ± 2.24ª	21.42 ± 3.71ª	21.04 ± 2.10ª
**TG (mg/dL)**	25.34 ± 7.75	30.25 ± 7.76ª	26.25 ± 5.28ª	23.92 ± 4.68ª	26.13 ± 4.57ª	30.27 ± 4.63ª

C (normocholesterolemic group), HC (hypercholesterolemic group), AQ 70 (HC + aqueous extract 70 mg/kg), AQ 140 (HC + aqueous extract 140 mg/kg), NEPF 7 (HC + non-esterified phenolic fraction 7 mg/kg), NEPF 14 (HC + non-esterified phenolic fraction 14 mg/kg).

The results are expressed as the means ± S.E.M (n = 5-8).

Different letters represent significant differences between rows (Tukey-Kramer test, p < 0.05), * significant difference between normocholesterolemic (C) *vs* hypercholesterolemic (HC) groups by Student’s t test (p < 0.05).

### Effect of the aqueous extract and non-esterified phenolic fraction on serum lipids

The TC and non-HDL-c levels were increased in the serum of rats submitted to a hypercholesterolemic diet (HC group) compared with the normocholesterolemic (C) group (p < 0.001). These results are similar to those reported in the literature using the same experimental model [10,13]. Moreover, the HC group had lower serum levels of HDL-c than the C group (p < 0.001, Table 3). Although both concentrations of the NEPF did not have a significant effect on the serum lipid profile, the AQ at 70 mg/kg significantly reduced the serum TC and non-HDL-c levels by 39.8% (p < 0.01) and 44.4% (p < 0.05), respectively, compared with the HC group but showed no effects on the TG and HDL-c levels. The AQ at a higher concentration (140 mg/kg body weight) did not result in any substantial reduction in the serum TC and non-HDL-c levels (p > 0.05 AQ70 *vs* AQ140, Table 3).

### Effect of the aqueous extract and non-esterified phenolic fraction on thiobarbituric acid reactive substances (TBARS)

**Figure 1:**
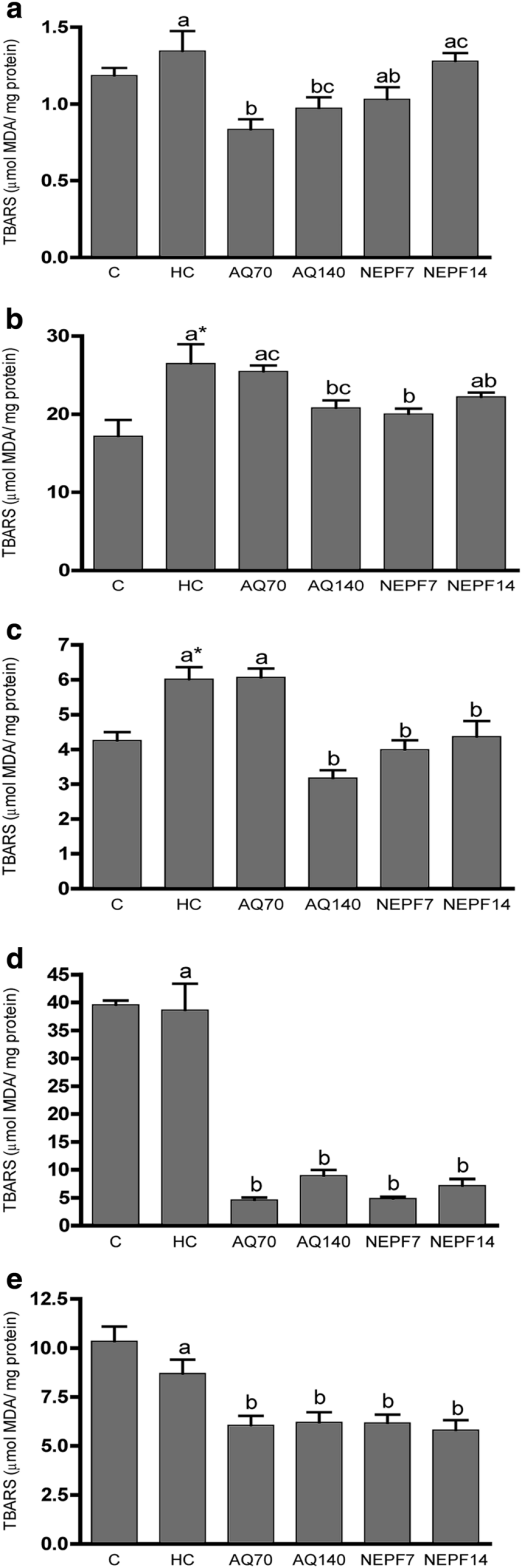
**TBARS concentrations (μmol MDA/mg protein) in the serum, heart, kidneys, brains and liver. **C (normocholesterolemic group), HC (hypercholesterolemic group), AQ 70 (HC + aqueous extract 70 mg/kg), AQ 140 (HC + aqueous extract 140 mg/kg), NEPF 7 (HC + non-esterified phenolic fraction 7 mg/kg), NEPF 14 (HC + non-esterified phenolic fraction 14 mg/kg). The results are expressed as the means ± S.E.M; n = 5–8. Different letters represent significant differences between groups (Tukey-Kramer test, p < 0.05). * p < 0.05 normocholesterolemic (C) *vs* hypercholesterolemic (HC) groups by Student’s t test.

In addition to an effect on the lipid profile, AQ at 70 mg/kg body weight was also able to significantly reduce the serum TBARS levels from 1.34 to 0.83 μmol MDA/mg protein compared with the untreated hypercholesterolemic rats (Figure 1a). Regarding the tissue TBARS levels, an increase in lipid peroxidation was observed in the HC group compared with the normocholesterolemic group in the heart and kidneys (Figure 1b and c, respectively). The NEPF at 7 and 14 mg/kg was able to reduce the TBARS levels in the heart (24.32% and 16.05%, respectively) and renal tissues (33.61 and 27.29%, respectively).

In the brain and liver (Figure 1d and e, respectively), no difference was observed in the HC group compared with the C group. However, the AQ and NEPF groups notably showed lower TBARS levels compared with the HC group in these tissues.

### Activity of antioxidant enzymes

**Figure 2:**
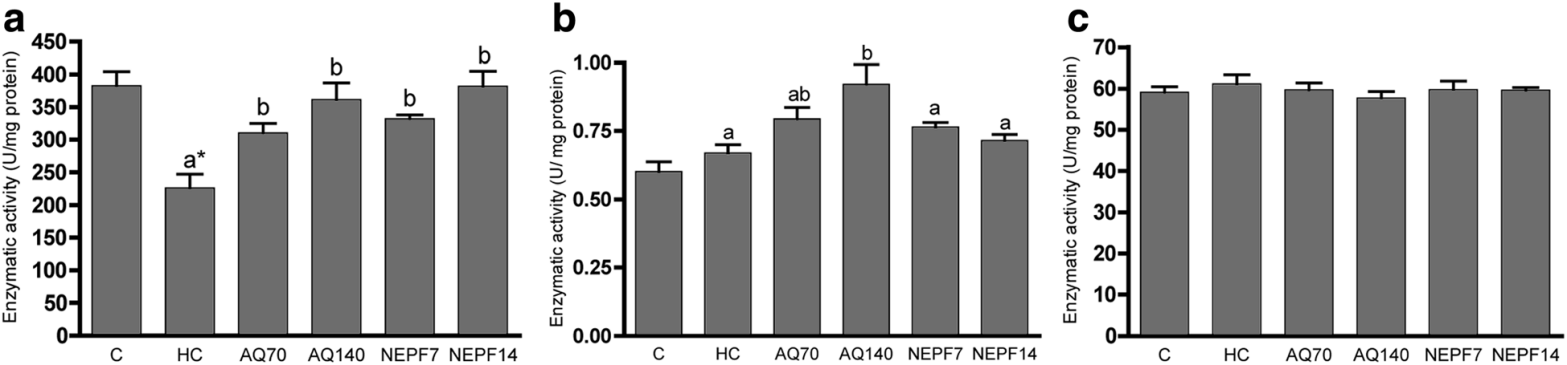
**Activity of the antioxidant enzymes catalase (a), glutathione peroxidase (b) and superoxide dismutase (c) in the liver. **C (normocholesterolemic group), HC (hypercholesterolemic group), AQ 70 (HC + aqueous extract 70 mg/kg), AQ 140 (HC + aqueous extract 140 mg/kg), NEPF 7 (HC + non-esterified phenolic fraction 7 mg/kg), NEPF 14 (HC + non-esterified phenolic fraction 14 mg/kg). The results are expressed as the means ± S.E.M; n = 5–8. Different letters represent significant differences between groups (Tukey-Kramer test, p < 0.05). * p < 0.05 normocholesterolemic (C) *vs* hypercholesterolemic (HC) groups by Student’s t test.

**Figure 3:**
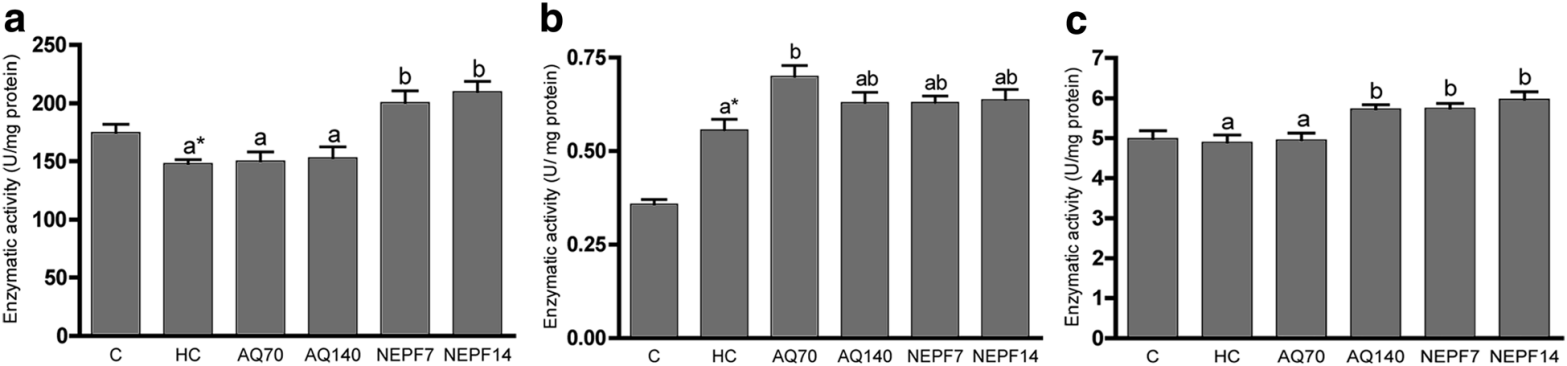
**Activity of the antioxidant enzymes catalase (a), glutathione peroxidase (b) and superoxide dismutase (c) in the kidneys. **C (normocholesterolemic group), HC (hypercholesterolemic group), AQ 70 (HC + aqueous extract 70 mg/kg), AQ 140 (HC + aqueous extract 140 mg/kg), NEPF 7 (HC + non-esterified phenolic fraction 7 mg/kg), NEPF 14 (HC + non-esterified phenolic fraction 14 mg/kg). The results are expressed as the means ± S.E.M; n = 5–8. Different letters represent significant differences between groups (Tukey-Kramer test, p < 0.05). * p < 0.05 normocholesterolemic (C) *vs* hypercholesterolemic (HC) groups by Student’s t test.

**Figure 4:**
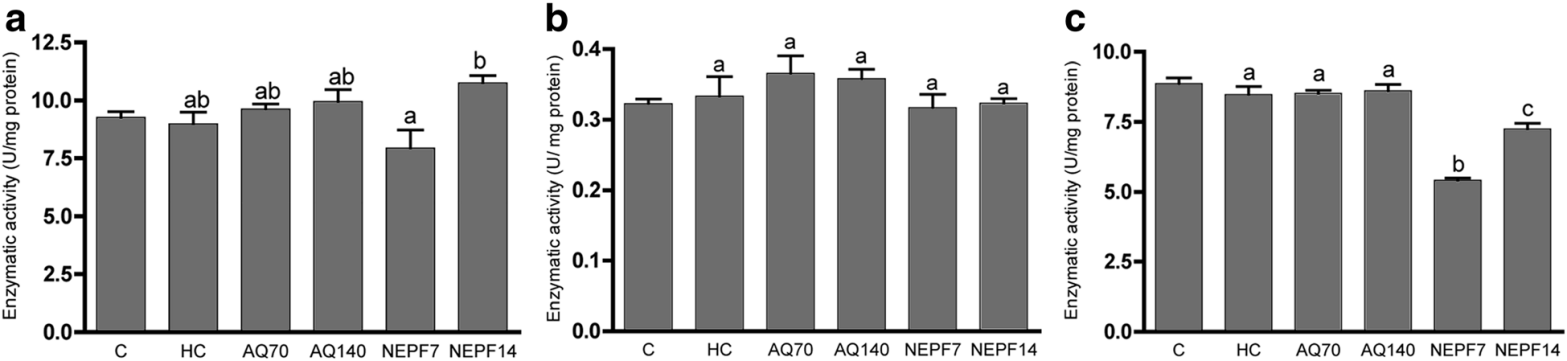
**Activity of the antioxidant enzymes catalase (a), glutathione peroxidase (b) and superoxide dismutase (c) in the heart. **C (normocholesterolemic group), HC (hypercholesterolemic group), AQ 70 (HC + aqueous extract 70 mg/kg), AQ 140 (HC + aqueous extract 140 mg/kg), NEPF 7 (HC + non-esterified phenolic fraction 7 mg/kg), NEPF 14 (HC + non-esterified phenolic fraction 14 mg/kg). The results are expressed as the means ± S.E.M; n = 5–8. Different letters represent significant differences between groups (Tukey-Kramer test, p < 0.05). * p < 0.05 normocholesterolemic (C) *vs* hypercholesterolemic (HC) groups by Student’s t test.

Although the liver CAT activity was significantly lower in the HC rats than in the C rats (226.30 ± 20.68 *vs* 382.60 ± 21.88 U/mg protein), the oral administration of both phenolic extracts from rosemary at the lower concentration was able to significantly increase (p < 0.05) the CAT activity, and this effect was more prominent with the higher concentration (p < 0.001), thereby showing a dose–response effect (Figure 2a). With respect to GPx, only the highest concentration of the AQ was able to significantly increase the activity of this enzyme (Figure 2b). However, no effect was observed for SOD activity in either of the treatments (Figure 2c). As observed in the hepatic tissue, the CAT activity in the kidneys of the HC group was significantly lower than in the C group, but the NEPF increased the CAT and SOD activities at both concentrations (Figure 3a and c, respectively). In cardiac tissue, none of the antioxidant enzymes presented a difference in their activities among the C and HC groups (p > 0.05). The AQ did not exert a significant effect on the activities of the antioxidant enzymes. In contrast to the data obtained from the kidneys, NEPF was not able to increase the SOD and CAT cardiac activities (Figure 4a and c, respectively).

**Figure 5:**
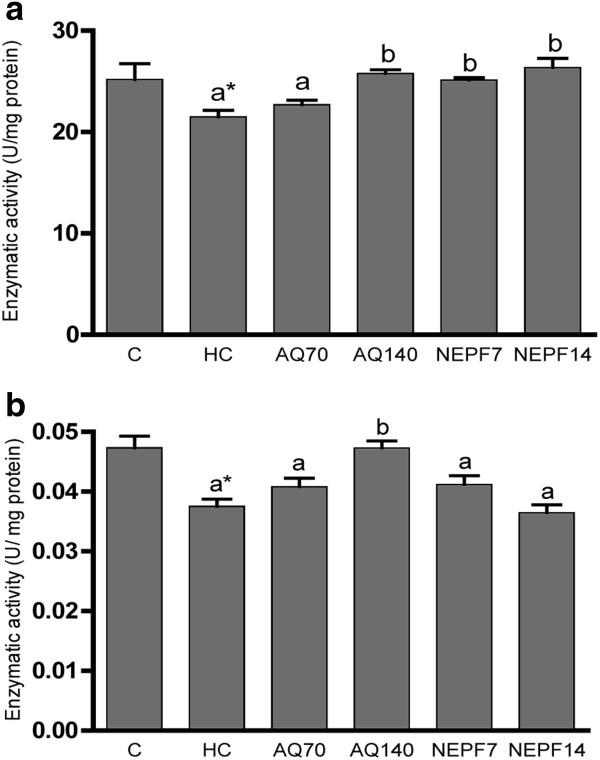
**Activity of the antioxidant enzymes superoxide dismutase (a) and glutathione peroxidase (b) in the brain. **C (normocholesterolemic group), HC (hypercholesterolemic group), AQ 70 (HC + aqueous extract 70 mg/kg), AQ 140 (HC + aqueous extract 140 mg/kg), NEPF 7 (HC + non-esterified phenolic fraction 7 mg/kg), NEPF 14 (HC + non-esterified phenolic fraction 14 mg/kg). The results are expressed as the means ± S.E.M; n = 5–8. Different letters represent significant differences between groups (Tukey-Kramer test, p < 0.05). * p < 0.05 normocholesterolemic (C) *vs* hypercholesterolemic (HC) groups by Student’s t test.

The hypercholesterolemic group had a significant decrease in the SOD and GPx activities, indicating oxidative damage in the brain. As observed in the liver, only the highest AQ concentration was able to increase the activities of the above enzymes (p < 0.001). The NEPF did not raise the GPx activity (Figure 5b) but was able to increase the SOD activity (14.45% for 7 mg/kg and 18.41% for 14 mg/kg, p < 0.001).

### Fatty acid profile of hepatic tissue

Table 4 shows that the hypercholesterolemic diet increased the total lipids and led to a modification of the lipid composition in the liver, as previously reported [6,32]. No significant difference in the amount of total lipids, SAFA, MUFA and PUFA was observed between the treated and untreated hypercholesterolemic groups. However, a reduction in the amount of saturated fatty acids (SAFA, p < 0.001) and an increase in monounsaturated fatty acids (MUFA, p < 0.001) occurred in the hypercholesterolemic group compared with the normocholesterolemic group (Table 4).

**Table 4:** Fatty acid composition (g/100 g fatty acids) of liver

	**C**	**HC**	**AQ 70**	**AQ 140**	**NEPF 7**	**NEPF 14**
**C16:0**	20.36 ± 1.96	13.77 ± 0.54^ab*^	13.84 ± 0.36^ab^	14.76 ± 1.52^b^	13.11 ± 0.51^a^	12.58 ± 0.59^a^
**C18:0**	18.27 ± 2.22	7.20 ± 1.28*	6.66 ± 0.58	6.10 ± 1.00	6.22 ± 0.95	6.24 ± 0.84
**Total SAFA**	**39.87 ± 3.13**	**21.49 ± 1.67**^a*^	**20.90 ± 0.73**^**ab**^	**21.39 ± 1.63**^**a**^	**20.59 ± 1.43**^**ab**^	**19.14 ± 0.88**^**b**^
**C16:1**	0.64 ± 0.37	3.57 ± 0.85^ab*^	3.17 ± 0.60^a^	2.74 ± 0.47^a^	3.76 ± 0.79^ab^	3.57 ± 0.61^b^
**C18:1**	11.75 ± 4.20	25.12 ± 1.78^*^	25.47 ± 1.12	26.18 ± 1.66	25.50 ± 1.93	26.37 ± 1.73
**Total MUFA**	**12.48 ± 4.66**	29.38 ± 2.48^*^	**29.14 ± 1.73**	**29.23 ± 1.77**	**29.76 ± 2.69**	**30.37 ± 1.96**
**C18:2**	20.29 ± 3.79	31.42 ± 0.85^*^	32.33 ± 0.77	32.08 ± 2.05	31.57 ± 1.47	32.30 ± 1.26
**Total PUFA**	**45.85 ± 6.55**	**47.54 ± 1.08**	**49.20 ± 1.19**	**48.59 ± 2.34**	**47.77 ± 1.89**	**48.05 ± 1.96**
**C18:1/C18:0**	0.64	3.49	3.82	4.29	4.10	4.23
**Total lipids (mg)**	**11.71 ± 2.51**	25.06 ± 5.46^*^	**26.09 ± 4.62**	**28.13 ± 3.56**	**25.77 ± 4.93**	**26.49 ± 1.65**

C (normocholesterolemic group), HC (hypercholesterolemic group), AQ 70 (HC + aqueous extract 70 mg/kg), AQ 140 (HC + aqueous extract 140 mg/kg), NEPF 7 (HC + non-esterified phenolic fraction 7 mg/kg), NEPF 14 (HC + non-esterified phenolic fraction 14 mg/kg).

SAFA: Saturated Fatty Acids; MUFA: Monounsaturated Fatty Acids; PUFA: Polyunsaturated Fatty Acids.

The results are expressed as the means ± S.E.M (n = 5-8).

Different letters represent significant differences between groups (Tukey-Kramer test, p < 0.05).

* significant difference between normocholesterolemic (C) *vs* hypercholesterolemic (HC) groups by Student’s t test (p < 0.05).

## Discussion

In this investigation, we performed *in vivo* experiments that showed that only AQ 70 was capable of significantly reducing the serum TC and non-HDL-c levels in diet-induced hypercholesterolemic rats, as demonstrated in Table 3. This effect may be attributed to the higher amount of total phenolic compounds found in AQ, which was almost twice as much as in NEPF, which represented a more purified fraction. This result suggests that other phenolic compounds, beyond those found in the NEPF, may be responsible for these responses. This effect on cholesterol reduction may be attributed to a decrease in the micellar solubilization of cholesterol in the digestive tract, to an increase in bile flow, bile cholesterol and bile acid concentration and to a subsequent increase in the fecal excretion of steroids, as previously described [33,34].

This study showed that both extracts (AQ70, AQ140) obtained from rosemary decreased the tissue TBARS levels, lowering the susceptibility to the peroxidative damage elicited by a cholesterol-rich diet. This effect could be attributed to the antiradical activity of these extracts, as demonstrated by the DPPH^●^ assay. In this context, AQ70 could contribute to a decrease in the CVD risk because it simultaneously reduced the TBARS levels and improved the plasma lipid contents.

This protection could be attributed to rosmarinic acid, which has been reported to inhibit the expression of inducible nitric oxide synthase (iNOS) and suppress the production of superoxide and 3-nitrotyrosine in Raw 264.7 macrophages [35].

Notably, oxidative stress is characterized by not only increased free radical production but also reduced antioxidant enzyme activities [7]. In fact, we observed that animals submitted to a high cholesterol diet plus water presented lower catalase activity in their hepatic and renal tissues (Figures 2a and 3a, respectively) compared with the animals receiving only a chow diet. An important observation from this study was that the phenolic compounds from rosemary were able to reestablish catalase activity, as observed with the AQ and NEPF in the liver and with the NEPF in the kidney. Data from the literature demonstrated that carnosic acid (found in NEPF) increased the antioxidant enzyme activities and inhibited lipid peroxidation in Caco-2 cells [36]. In kidneys, AQ70 also increased the GPx activity, which could be an adaptive process to cope with the free radical production (Figure 1c). However, AQ140 promoted a significant increase in the SOD activity (p < 0.05), similar to that observed for phenolic extracts from olive mill wastewater in which the antioxidant properties were only observed when administered at a higher concentration [10].

Both extracts (AQ and NEPF) at different concentrations were not able to increase the antioxidant enzyme activities in the cardiac tissue of the animals studied. Moreover, NEPF (7 mg/kg body weight) reduced the SOD and CAT activities. Bouderbala et al. [37] have also reported a reduction in SOD activity in the kidneys of hypercholesterolemic animals fed on a high cholesterol diet supplemented with *Ajuga iva* (0.5%). The observed effect was attributed to the lack of superoxide anion accumulation in these tissues. Extrapolating these results to our study, we can infer that the phenolic constituents of NEPF may have reduced the concentration of superoxide anions in the cardiac tissue, thereby reducing the need to activate the antioxidant enzymes.

The HC group had a significant decrease in the SOD and GPx activities, indicating oxidative stress in the brain. As observed for the kidneys, only the highest AQ concentration was able to increase the activities of these enzymes (p < 0.05), whereas NEPF was able to increase the SOD activity (14.95% for 7 mg/kg and 17.95% for 14 mg/kg, P < 0.05). A recent study showed that pre-treatment with an aqueous extract from a plant also belonging to the *Lamiaceae* family was able to reduce the cerebral infarct size and lipid peroxidation [38]. These protective effects involve the permeability of phenolic compounds across the blood–brain barrier, which has been demonstrated in both *in vitro* and *in situ* models [39,40].

Cholesterol-enriched diets also favor liver fat deposition, which plays a role in lipoprotein synthesis and metabolism and therefore in the cardiovascular disease risk [6,41]. In the present study, the increase in the total lipids and modification of the lipid composition in the HC group may be attributed to the higher stearoyl-CoA desaturase (SCD) activity elicited by cholesterol- and cholic acid-containing diets. In fact, in our study all animals fed on a hypercholesterolemic diet exhibited a higher oleic-to-stearic-acid ratio compared with the control group, which suggests a higher SCD activity as demonstrated in another study performed with the same animal model [42]. This effect may be a protective mechanism as SCD can convert saturated into monounsaturated fatty acids, which are preferred substrates for acyl-CoA:cholesterol acyltransferase (ACAT), an enzyme that catalyzes the esterification of hepatic free cholesterol to an inert cholesteryl ester [43]. However, no significant differences between the treated and untreated hypercholesterolemic groups were observed with respect to hepatic lipids. In this context, the effects of the phenolic compounds from rosemary on the lipoprotein levels can be suggested to be especially related to intestinal cholesterol absorption.

## Conclusion

In conclusion, our results suggest that phenolic compounds from *Rosmarinus officinalis* protect against hypercholesterolemia-induced oxidative stress, increasing the activities of antioxidant enzymes and reducing the amount of thiobarbituric acid reactive substances. The aqueous extract was also able to improve the serum lipid profile, contributing to cardiovascular disease reduction. Future analysis involving the bioavailability of rosemary phenolic compounds and their role on modulating the signaling pathways activated under oxidative stress-related diseases may help to clarify the role of these antioxidant compounds in cholesterol homeostasis and parameters of oxidative stress.

## Abbreviations

DPPH: 2,2-diphenyl-1-picrylhydrazyl;TBARS: Thiobarbituric Acid Reactive Substances;AQ: Aqueous Extract;NEPF: Non-Esterified Phenolic Fraction;CAT: Catalase;GPx: Glutathione Peroxidase;SOD: Superoxide Dismutase;GAE: Gallic Acid Equivalent;HPLC: High Performance Liquid Chromatography;BHT: Butylated Hydroxytoluene

## Competing interests

The authors declare that they have no competing interests.

## Authors’ contributions

For experimental design: MSA and JMF; for data collection: MSA, AMOS, EBTC, DPR and RP; for data analysis: MSA, AMOS, EBTC, JMF, MMR, AML, SBMB and DPR; for drafting the manuscript: MSA, AMOS, DPR, MMR, AML, SBMB and JMF. All authors read and approved the final manuscript.
